# SCReadCounts: estimation of cell-level SNVs expression from scRNA-seq data

**DOI:** 10.1186/s12864-021-07974-8

**Published:** 2021-09-22

**Authors:** N. M. Prashant, Nawaf Alomran, Yu Chen, Hongyu Liu, Pavlos Bousounis, Mercedeh Movassagh, Nathan Edwards, Anelia Horvath

**Affiliations:** 1 McCormick Genomics and Proteomics Center, School of Medicine and Health Sciences, The George Washington University, Washington, DC, 20037 USA; 2 Departments of Genetics and Genomic Sciences, Icahn School of Medicine at Mount Sinai, New York, NY USA; 3 Department of Biochemistry and Molecular & Cellular Biology, Georgetown University, Washington, DC, 20057 USA; 4 Harvard T.H. Chan School of Public Health, Boston, MA 02115 USA; 5 Department of Data Sciences, Dana Farber Cancer Institute, Boston, MA USA

**Keywords:** SNV, SNP, scRNA-seq, Mutation, Somatic mutation, Allele, Allele expression, Single cell, Single cell RNA sequencing

## Abstract

**Background:**

Recent studies have demonstrated the utility of scRNA-seq SNVs to distinguish tumor from normal cells, characterize intra-tumoral heterogeneity, and define mutation-associated expression signatures. In addition to cancer studies, SNVs from single cells have been useful in studies of transcriptional burst kinetics, allelic expression, chromosome X inactivation, ploidy estimations, and haplotype inference.

**Results:**

To aid these types of studies, we have developed a tool, SCReadCounts, for cell-level tabulation of the sequencing read counts bearing SNV reference and variant alleles from barcoded scRNA-seq alignments. Provided genomic loci and expected alleles, SCReadCounts generates cell-SNV matrices with the absolute variant- and reference-harboring read counts, as well as cell-SNV matrices of expressed Variant Allele Fraction (VAF_RNA_) suitable for a variety of downstream applications. We demonstrate three different SCReadCounts applications on 59,884 cells from seven neuroblastoma samples: (1) estimation of cell-level expression of known somatic mutations and RNA-editing sites, (2) estimation of cell- level allele expression of biallelic SNVs, and (3) a discovery mode assessment of the reference and each of the three alternative nucleotides at genomic positions of interest that does not require prior SNV information. For the later, we applied SCReadCounts on the coding regions of *KRAS*, where it identified known and novel somatic mutations in a low-to-moderate proportion of cells. The SCReadCounts read counts module is benchmarked against the analogous modules of GATK and Samtools. SCReadCounts is freely available (https://github.com/HorvathLab/NGS) as 64-bit self-contained binary distributions for Linux and MacOS, in addition to Python source.

**Conclusions:**

SCReadCounts supplies a fast and efficient solution for estimation of cell-level SNV expression from scRNA-seq data. SCReadCounts enables distinguishing cells with monoallelic reference expression from those with no gene expression and is applicable to assess SNVs present in only a small proportion of the cells, such as somatic mutations in cancer.

**Supplementary Information:**

The online version contains supplementary material available at 10.1186/s12864-021-07974-8.

## Background

Single cell RNA sequencing (scRNA-seq) brings major advantages over bulk RNA-seq analyses, especially the ability to distinguish cell populations and to assess cell-type specific phenotypes [1]. Connecting these phenotypes to cell-level genetic variants (such as Single Nucleotide Variants, SNVs) is essential for phenotype interpretation. In cancer, studies on cellular genetic heterogeneity have been instrumental in tracing lineages and resolving sub-clonal tumor architecture [2–9] In addition to cancer studies, SNV observations from single cells have been useful in studies of transcriptional burst kinetics, allelic expression, chromosome X inactivation, ploidy estimations, haplotype inference, and quantitative trait loci (QTL) [10–21].

**Fig. 1:**
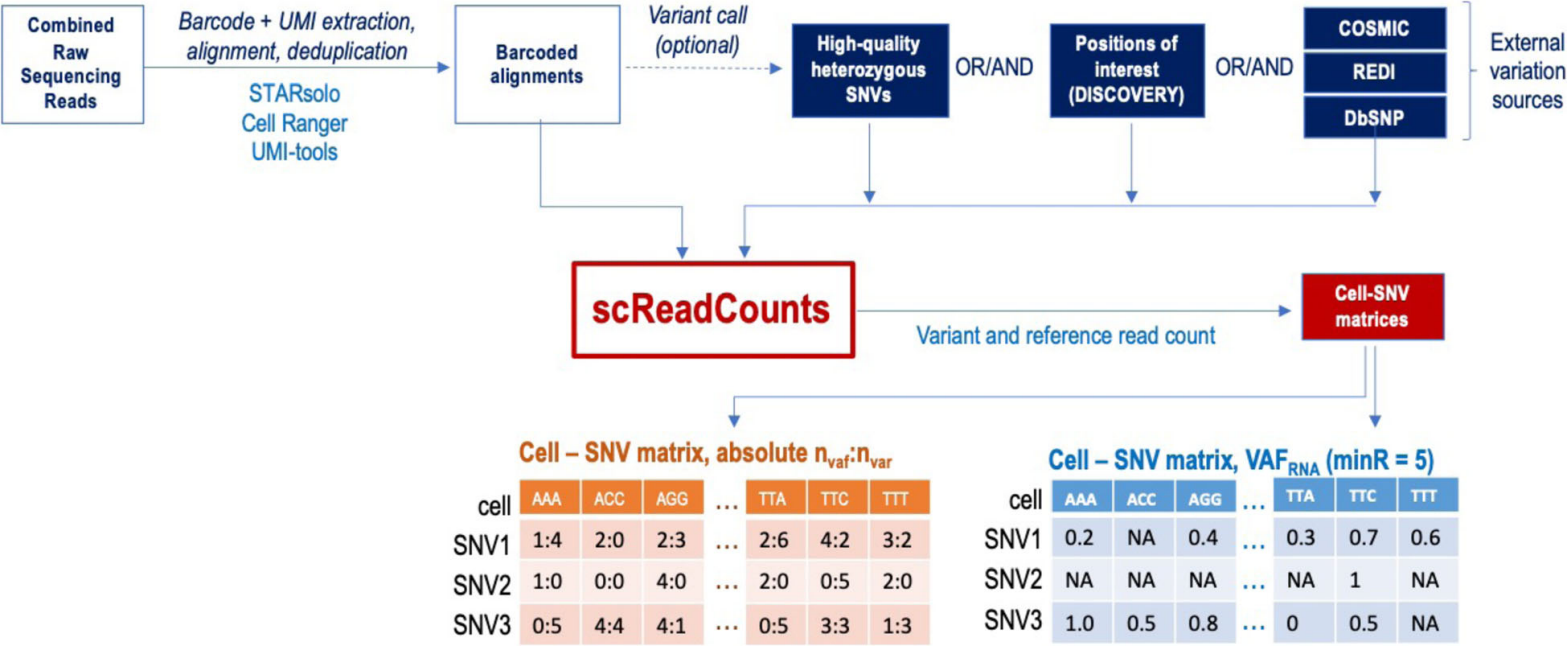
SCReadCounts workflow using publicly available tools

To aid with the considerable data-analysis and data-management demands of such studies, we have developed a tool, SCReadCounts, for cell-level quantitation of SNV expression. Provided with barcoded scRNA-seq alignments, list of barcodes and genomic loci and alleles of interest, SCReadCounts tabulates, for each cell, the reference and variant read counts (n_ref_ and n_var_, respectively), and expressed Variant Allele Fraction (VAF_RNA_ = n_var_/(n_var_ + n_ref_)). SCReadCounts generates a cell-SNV matrix with the absolute n_var_ and n_ref_ counts, and a cell-SNV matrix with the VAF_RNA_ estimated at a user-defined threshold of minimum number of required sequencing reads (minR) (Fig. 1). Particular strengths of SCReadCounts include its named, explicit, flexible, and configurable read-filtering and cell-barcode extraction capabilities; accounting for all reads overlapping each locus, whether counted or ignored; and straightforward input and output formats – these features make SCReadCounts easy to integrate in multi-tool analysis pipelines. The cell-SNV matrices then can be used for a wide range of downstream analyses.

Unlike variant callers (i.e. GATK, Samtools [22, 23]), SCReadCounts estimates the read counts per allele and per cell, across all cells, including cells in which the position of interest is covered with only reference reads. This is particularly useful in scRNA-seq settings, where it enables distinguishing cells with monoallelic reference expression from those with no gene expression. The later can be used to assess cell-level allele dynamics, and to correlate variant expression to gene expression [24].

### Implementation

SCReadCounts is freely available (https://github.com/HorvathLab/NGS) as a self-contained binary package for 64-bit Linux and MacOS (Darwin) systems, and as Python source. The self-contained binary packages are appropriate for most Linux and MacOS users. The pythonic version requires pysam, numpy and scipy along with other packages (detailed instructions at https://github.com/HorvathLab/NGS#SCReadCounts).

Currently, SCReadCounts has three programs. The program scReadCounts manages the sequential execution of programs readCounts and readCountsMatrix, collects the necessary arguments for successful execution, and avoids unnecessary execution of the expensive readCounts tool if possible. readCounts requires three input files: a pooled single cell alignment, a list of cell-barcodes, and a list of genomic positions of interest. Optionally, readCounts can be user-configured for read filtering. readCounts utilizes the barcode information from the pooled single cell alignments and outputs the variant and reference read counts (n_var_ and n_ref_, respectively), for each barcode (cell), in a tab separated text file. This file is then used as an input for the second program - readCountsMatrix - which, upon providing an output prefix, generates two outputs: (1) a cell-position matrix with absolute n_var_ and n_ref_ counts, and (2) a cell-position matrix with the expressed VAF_RNA_. VAF_RNA_ is estimated at a user-defined threshold of minimum required sequencing reads (minR); default minR = 5. readCountsMatrix is time-efficient and can be re-run multiple times at various minR thresholds. 

SCReadcounts provides explicit configuration for alignments barcoded through STARsoloUMItools. Additional cellular barcode extraction logic can be configured for SCReadCounts software, based on BAM file tags or RNA sequence name and delimited tokens or regular expressions (see the “SCReadCounts Read Grouping” documentation).

#### Performance

**Fig. 2:**
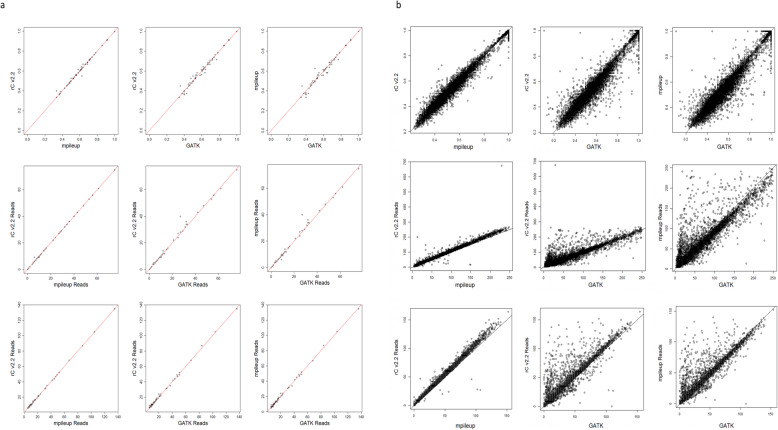
Concordance between n_var_, n_ref_ and VAF_RNA_ estimates across SCReadCounts, the mpileup utility of Samtools, and the haplotype caller of GATK in individual cell scRNA-seq alignments (**a**) and pooled scRNA-seq alignments (**b**) of sample. SRR10156295. Top: VAF_RNA_ middle: n_var_, bottom: n_ref._

 To assess SCReadCounts performance we compared the variant and reference read counts tabulations of SCReadCounts with the analogous modules of the mpileup utility of Samtools and the haplotype caller of GATK [22, 23]. SCReadCounts default options generate nearly identical values to mpileup and GATK (Fig. 2). SCReadCounts uses, by default, a very simple read-filtering criteria, but it can also be readily configured to achieve scenario-specific, mpileup-consistent, or GATK-consistent results, with optional explicit output of the number of reads discarded by each filtering rule. On our system (2 × 14 core CPUs with 1.5 TB RAM compute node) processing of a file containing ~ 5000 cells, ~150mln reads, and ~ 80 K SNVs, requires approximately 4 h for the tabulation of n_var_ and _nref,_ and up to 2 min for the generation of the cell-SNV matrices. The later enables the users to quickly generate VAF_RNA_ matrices at various minR.

## Results

We have explored a variety of SCReadCounts applications on over 300,000 single cells from six different studies on normal and tumor human samples, including adipose tissue, adrenal neuroblastoma, acute myeloid leukemia, non-small lung cancer, prostate carcinoma, and the MCF7 cell line derived from breast adenocarcinoma [3–6, 25–27]. Here we demonstrate three different SCReadCounts applications on 59,884 cells derived from seven neuroblastoma samples [3]: (1) estimation of cell level expression of known somatic mutations and RNA-editing sites, (2) estimation of cell level allele expression from biallelic positions as called in the pooled scRNA-seq data, and (3) a discovery mode assessment of the reference and each of the three alternative nucleotides at genomic positions of interest. The discovery mode does not require prior knowledge on existing genetic variants and is particularly convenient for a quick focused assessment of a gene or a group of genes or regions of interest.

For all three applications the scRNA-seq data was processed using publicly available tools. In the exemplified workflow (See Fig. 1), the raw sequencing reads are aligned to the reference human genome (GRCh38) using STARsolo (v.2.7.7a) which processes the cellular barcodes, generates a list of error corrected barcodes, and deduplicates the alignments, retaining the reads with the highest mapping quality using the unique molecular identifiers (UMI) [28]. In addition to STARsolo, SCReadCounts accepts barcoded alignments and barcode lists generated by UMI-tools [29, 30]. The alignments can be filtered to correct for allele-mapping bias by removing reads mapped ambiguously due to the variant nucleotide (WASP); this filtering utilizes the same list of positions to be used as input for SCReadCounts [31].

### SCReadCounts on known variant loci

**Fig. 3:**
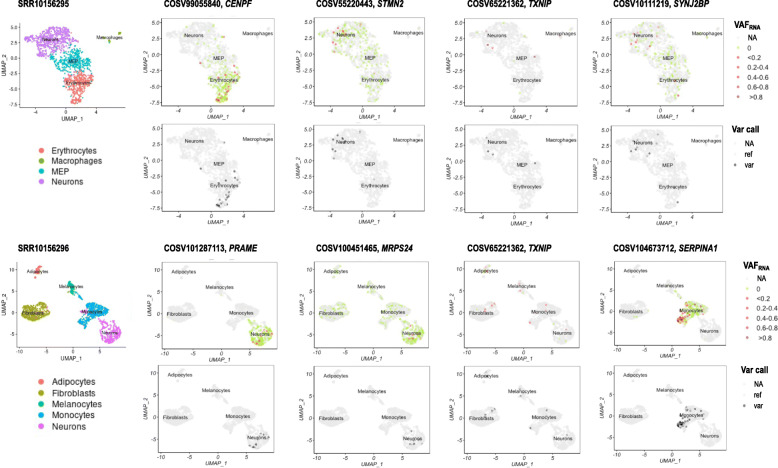
Two-dimensional UMAP clusters of samples SRR10156295 and SRR10156296 showing cells classified by type (left) and visualizing cell-level expression of somatic mutations (right): COSV99055840 in *CENPF*, COSV55220443 in *STMN2*, COSV85221362 in *TXNIP*, COSV10111219 in *SYNJ2BP*, COSV101287113 in *PRAME*, COSV100451465 in *MRPS24*, and COSV104673712 in *SERPINA1*. Each pair of panels shows VAF_RNA_, (top) where the intensity of the red color corresponds to the relative expression of the somatic mutation and the green color indicates that all the reads (minR = 3) covering the position in the cell carried the reference nucleotide, compared to variant call (bottom), where the black color indicates presence of the mutation, and grey indicates either lack of variant or lack of gene expression. Hence, SCReadCounts allows to distinguish cells with only reference reads from cells with low or no expression of the SNV-harboring gene

SCReadCounts can be applied to assess known genetic variation loci such as somatic mutational hotspots or RNA-editing sites. We first asked if we could assess known somatic mutations in the set of seven neuroblastoma scRNA-seq samples (S_Table 1). Known somatic mutations (118,401 loci) were extracted from COSMIC by selecting loci from the Cancer Census Genes positioned outside repetitive regions (to exclude alignment bias) and not present in dbSNP (to ensure germline variants are excluded (dbSNPv.154) [32]. The resulting list of COSMIC somatic mutations (S_Table 2) was provided to SCReadCounts. A minimum of 3 sequencing reads (n_var_ + n_ref_ > = 3, minR = 3) was required for loci to be considered for further analysis. SCReadCounts identified 450 distinct COSMIC mutations expressed in at least 4 individual cells in one or more of the 7 neuroblastoma samples (S_Table 3). Examples include COSV99055840 in *CENPF*, COSV55220443 in *STMN2*, COSV85221362 in *TXNIP*, COSV10111219 in *SYNJ2BP*, COSV101287113 in *PRAME*, COSV100451465 in *MRPS24*, and COSV104673712 in *SERPINA1* (Fig. 3). To assess the consistency between SCReadCounts and the variant callers across all cells, we split the alignments based on cell barcodes [33] and performed variant call on all the individual cell alignments using GATK in samples SRR10156295, SRR10156296, SRR10156299 and SRR10156300. GATK called all the SNVs for which 2 or more reads bearing the variant nucleotide were tabulated by scReadCounts (relaxed filtering, See 1) in the corresponding cells (See Fig. 3). Importantly, because SCReadCounts excludes loci not covered with required number of reads (minR), it allows to distinguish cells with only reference reads from cells with low or no expression of the SNV-harboring gene (See Fig. 3). Accordingly, based on the relative expression of variant reads and the SNV locus, SCReadCounts can discern different gene-SNV-expression patterns. For example, some genes and their harbored SNV are expressed primarily in one cell type (i.e. COSV99055840 in *CENPF* in erythrocytes, COSV101287113 in *PRAME* in neurons, and COSV104673712 in *SERPINA1* in monocytes, See Fig. 3). Other SNVs are expressed primarily in one cell type even when the gene is expressed across different cell types (i.e COSV55220443 in *STMN2 and* COSV10111219 in *SYNJ2BP* in neurons, see Fig. 3).

Next, we demonstrate that SCReadCounts can quantify cell-specific RNA-editing in the same neuroblastoma samples. For this analysis we used the previously described single nucleotide RNA-editing events catalogued in the REDI database [34], after excluding genomic positions in repetitive regions or that coincide with a potential germline variant. A total of 107,053 distinct RNA-editing sites were provided to SCReadCounts (S_Table 4) along with the corresponding scRNA-seq alignments. At minR = 5, SCReadCounts identified 72 positions which were edited in at least 2 cells in one or more of the 7 neuroblastoma samples (S_Table 5). We investigated the A > G RNA-editing event at 14:100846310 in the cancer-implicated lincRNA *MEG3*; this position was edited in 6 of the 7 samples. Cells with *MEG3* RNA-editing were predominantly clustered in neurons, where the proportion of edited RNA molecules (as reflected through the VAF_RNA_), suggested variable degrees of RNA editing (S_Figure 2).

#### SCReadCounts after variant call

SCReadCounts can be applied in conjunction with variant callers to estimate the cell-specific allele expression of germline or somatic SNVs. To explore this application, we called variants from the pooled alignments using GATK (v4.1.7.0, [22]), and filtered the calls retaining high quality biallelic positions for which both the variant and the reference allele were supported by a minimum of 50 sequencing reads, as we have previously described [24, 26]. The variant lists were then provided to SCReadCounts together with the corresponding alignments and the STARsolo generated list of error corrected barcodes. The resulting VAF_RNA_ estimates can be used to explore a variety of cell-level allelic features. For example, the distribution of the VAF_RNA_ at minR = 5 across the cells for each of the seven neuroblastoma samples is plotted on S_Figure 3, which shows that many of the SNVs have skewed or mono-allelic expression. VAF_RNA_ estimates for positions covered by at least 10 total reads (minR = 10) in 20 and more cells per sample are summarized in S_Table 6. A systematic analysis of the distribution VAF_RNA_ of heterozygous SNVs at different minR is analyzed in our recent work [26]. In addition, VAF_RNA_ estimates can be used to explore correlations between allele- and gene-expression in single cells using scReQTL. Our previous research applying scReQTL on normal adipose datasets has shown that scReQTLs are substantially enriched in GWAS-significant SNVs and in known gene-gene interactions [24].

#### SCReadCounts in discovery mode

**Fig. 4:**
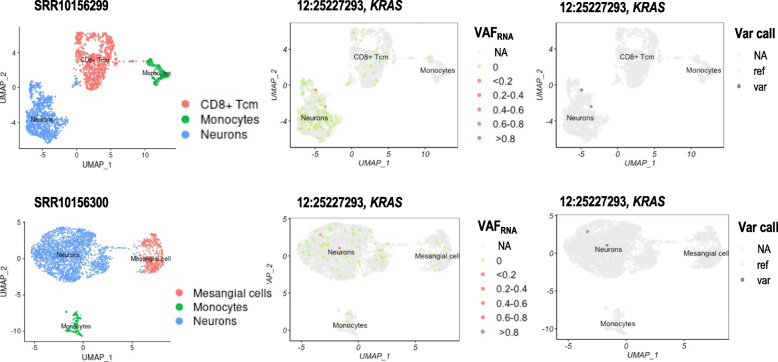
Two-dimensional UMAP clusters of samples SRR10156299 and SRR10156300 showing cells classified by type (left panel) and visualizing cell-level expression of the novel somatic mutation Gly77Gly (12:25227293_G > A) in the gene *KRAS* (middle panel) in comparison with results from variant call in barcode-split individual alignments (12:25227293_G > A – positive cells are indicated in black).

As mentioned earlier, SCReadCounts can be applied in a discovery mode which does not require any prior knowledge of SNVs. In this use-case, SCReadCounts considers positions of interest where the reference nucleotide is substituted with each of the three alternative nucleotides. Such SCReadCounts inputs can be generated for a gene, region or a group of genes/regions of interest, either manually, or using a script (provided at https://github.com/HorvathLab/NGS#SCReadCounts). Herein, we demonstrate this approach using an enumeration of each position in the coding region of *KRAS* (S_Table 7), mutations in which have been implicated in neuroblastoma. Across the seven samples, SCReadCounts identified a total of 30 distinct SNVs that do not coincide with known germline variants in the coding sequence of *KRAS* (S_Table 8). The SNVs included missense, nonsense and synonymous substitutions in up to 15 individual cells per sample. Eight of the 30 SNVs were seen in more than one sample; for example, the synonymous substation 12:25227293_G > A (Gly77Gly, Fig. 4) was seen in 4 out of the 7 samples. Seven of the 30 mutations were previously catalogued in the COSMIC database – the remaining 23 substitutions represent novel *KRAS* variants. To assess the consistency of SCReadCounts with variant calls, we applied the above-described strategy of GATK variant call on barcode-split individual cell alignments. Similarly to the COSMIC mutations, GATK called all the SNVs for which 2 or more reads bearing the variant nucleotide were tabulated by scReadCounts in the corresponding cells (See Fig. 4). In addition, we examined the IGV visualization of the alignments from 12:25227293_G > A positive cells; alignments from cells with different proportion of variant reads are shown on S_Figure 4.

## Discussion

SCReadCounts joins the handful of currently emerging approaches for analysis of genetic variation from scRNA-seq data [7, 8, 16, 35–37].

SCReadCounts supports several use-cases specific to scRNA-seq. First, and perhaps most importantly, SCReadCounts can detect and quantify SNVs present in a low proportion of cells, such as somatic and mosaic SNVs. SNVs present in a low proportion of cells are known to be frequently missed by variant calls carried out on pooled scRNA-seq alignments [35, 36, 38]. Indeed, the majority of the somatic mutations detected by SCReadCounts were not called on the pooled scRNA-seq data by GATK at its default setting but were called after extracting individual cell alignments by barcode (See S_Tables 3, 8). Splitting alignments by barcodes and calling variants from thousands of individual cells however is computationally expensive [39]. The statistical significance of novel cell-level somatic mutations can be readily assessed by tools such as SCmut [35] from SCReadCounts matrices, when the experimental design and availability of bulk sequencing data permit. However, even without such sophisticated approaches, SCReadCounts minR criteria provides some measure of control over false positives in discovery mode, and surfaces read counts for cells with only reference reads. Thereby SCReadCounts enables exploration of gene-SNV expression patterns and outlining SNVs specific for a cell type for cell-specific or ubiquitously expressed genes (See Fig. 3).

In the herein presented analysis, SCReadCounts detected novel somatic mutations occurring in individual cells or in a small number of cells in one or more of the neuroblastoma samples. Second, SCReadCounts provides per cell quantitation of absolute and relative number of variant reads across all cells, including those where the position is covered with only reference reads, allowing the identification of cells with preferential expression of the variant or the reference allele. Furthermore, the flexible VAF_RNA_ minR enables tuning of SCReadCounts to the particular application and depth of sequencing. Here, a major consideration is the balance between inclusivity (low minR) and higher-confidence VAF_RNA_ estimates (high minR). In our analyses we use different minR depending on the application [24, 26]. For example, to confidently estimate allele expression of germline variants in highly transcribed genes, high minR is needed. In contrast, assessments of somatic mutations would benefit from high inclusivity using low minR. Finally, SCReadCounts generates cell-SNV matrices that are analogous to the cell-gene matrices generated by popular scRNA-seq tools, which streamlines down- stream applications combining SNV and gene expression.

## Conclusions

In conclusion, we believe that SCReadCounts supplies a fast and efficient solution for estimation of scRNA-seq genetic variation. Importantly, SCReadCounts enables distinguishing cells with monoallelic reference expression from those with no gene expression and is applicable to assess SNVs present in only a small proportion of the cells, such as somatic mutations in cancer.

## Materials and methods

### Sequencing datasets

The sequencing datasets were freely available from the NCBI Sequence Read Archive (SRA) under the accession numbers SRR10156295, SRR10156296, SRR10156297, SRR10156299, SRR10156300, SRR10156302, and SRR10156303. The patients demographics, neuroblastoma (NB) phenotype, obtaining of the samples, and the specifics of the 10x Genomics sequencing protocol are described in details in the original study [3]. Briefly, all the samples were processed using 10x Genomics 3-UTR v2 workflow, and the produced libraries were sequenced to a 150 nt sequencing length on NovaSeq 6000 (Illumina) at a depth of approximately 400 M reads per sample.

### Data processing

For alignment of the scRNA sequencing reads, read-to-gene assignment, cell barcode demultiplexing, error correction, and unique molecular identifier (UMI) collapsing, we used the STARsolo module of STAR v.2.7.7a with transcript annotations from the assembly GRCh38.79 [28] as previously described [24]. To generate individual cell alignments we adopted a publicly available python script which splits the pooled scRNA-seq alignments based on cellular barcode [40].

### Variant call

SNV were called using the HaplotypeCaller module of GATK v.4.2.0.0 [22], filtered using bcftools v.1.10.2 [23] and annotated using SeattleSeq v.16.00 (dbSNP build 154), as previously described [24]. When calling SNVs from the individual alignments, no filtering was applied on the SNV calls in order to retain calls where the variant nucleotide is present in a single read.

### Gene expression estimation from scRNA-seq data

Gene expression was estimated from raw read count matrices output by STARsolo, and normalized and scaled using the SCTransform function of Seurat v.3.0 [41, 42], as previously described [24, 43]. Based on the cells’ and features’ distribution, we have filtered out: (1) cells with mitochondrial gene expression over between 7.5 and 15%, (2) cells with less than between 500 and 1000 genes, and (3) cells with more than between 2600 and 4500 detected genes (to remove potential doublets, examples on S_Figure 5). Batch effects and cell cycle effects were removed as previously described [24].

### Cell type assessments

Likely cell types were assigned to cell clusters using SingleR v.1.0.5 [44] as previously described [24]; examples are shown on S_Figure 6.

### Statistical analyses

Statistical assessments were performed using the statistical modules implemented in the used software packages [41, 44], with built-in multiple testing corrections, where *p*-value of 0.05 was considered significant.

## Supplementary Information

**Additional file 1: Supplementary Fig. 1.** CCND1. Two-dimensional UMAP clusters of samples SRR10156296, SRR10156297 and SRR10156299 showing cells classified by type (left) and visualizing COSV57121427 in *CCND1*, which was seen primarily in neurons in all three samples. The intensity of the red color corresponds to the proportion variant reads) of the VAF_RNA_ of the indicated somatic mutations in the corresponding cells of the neuroblastoma samples; green color indicates that all the reads (minR = 3) covering the position in the cell carried the reference nucleotide.

**Additional file 2: Supplementary Fig. 2.** RNA-editing. Two-dimensional UMAP clusters of samples SRR10156297 and SRR10156299 showing cells classified by type (left) and visualizing RNA-editing levels (right) in the gene MEG3, where the intensity of the red color corresponds to the proportion of edited reads, and the green color indicates that all the reads (minR = 3) covering the position in the cell carried the reference nucleotide.

**Additional file 3: Supplementary Fig. 3.** scVAF_RNA_ distribution. scVAF_RNA_ estimated at genomic positions covered by a minimum of 5 sequencing reads (minR = 5) at the sites with bi-allelic calls (GATK) in the 7 neuroblastoma samples; the positions are sorted by VAFRNA (y-axis). For the majority of positions, VAF_RNA_ showed predominantly mono-allelic expression, with a substantial proportion of the scVAF_RNA_ estimations in the intervals 0–0.2 (orange) and 0.8–1.0 (purple). The percentage of cells with the corresponding VAF_RNA_ is displayed on the x-axis.

**Additional file 4: Supplementary Fig. 4.** KRAS. IGV visualization of variable scVAF_RNA_ of the novel somatic mutation Gly77Gly (12:25227293_G > A) in the gene *KRAS* in three individual cells of sample SRR10156295.

**Additional file 5: Supplementary Fig. 5.** Before and after filtering features distribution. Examples of density plots showing the distribution of cells based on proportion of transcripts of mitochondrial origin and number of genes, plotted against the counts of sequencing reads before (top) and after (bottom) filtering. The selected QC thresholds are: mitochondrial gene expression above between 6 and 15%, and number of genes below between 800 and 1000. To remove potential doublets/multiples we also filtered out signals with more than between 2600 and 4500 genes.

**Additional file 6: Supplementary Fig. 6.** SingleR Heatmaps. Heatmaps of SingleR scores for top correlated cell types from each of Seurat generated clusters. SingleR uses expression data to regenerate the clusters, and for each cluster, calculates the Spearman coefficient for the genes in the reference dataset. Then, it uses multiple correlation coefficient to collect a single value per cell type per cluster.

**Additional file 7: Supplementary Table 1.** Samples. Samples used in this study.

**Additional file 8: Supplementary Table 2.** COSMIC input. List COSMIC somatic mutations used as input to SCReadCounts.

**Additional file 9: Supplementary Table 3.** COSMIC output. 450 distinct COSMIC mutations found by SCReadCounts to be expressed in at least 4 individual cells in one or more of the 7 neuroblastoma samples.

**Additional file 10: Supplementary Table 4.** REDI input. 107,053 distinct RNA-editing sites provided as input to SCReadCounts.

**Additional file 11: Supplementary Table 5.** REDI output. 72 positions found by SCReadCounts to be edited in at least 2 cells in one or more of the 7 neuroblastoma samples.

**Additional file 12: Supplementary Table 6.** scVAF_RNA_ estimates. scVAF_RNA_ estimates for positions covered by at least 10 total reads (minR = 10) in 20 and more cells per sample.

**Additional file 13: Supplementary Table 7.** KRAS input in Discovery mode. Genomic positions used as a discovery mode input for scReadCounts.

**Additional file 14: Supplementary Table 8.** KRAS output in Discovery mode. 30 distinct SNVs that do not coincide with known germline variants in the coding sequence of KRAS.

## Data Availability

All the data analyzed in this study are supplied with the supplemental material or available as indicated in the cited publications. The sequencing datasets are downloaded from the NCBI Sequence Read Archive (SRA) under the accession numbers SRR10156295, SRR10156296, SRR10156297, SRR10156299, SRR10156300, SRR10156302, and SRR10156303.

## Abbreviations

scRNA-seq: Single cell RNA sequencing
SNV: Single nucleotide variant
SNP: Single nucleotide polymorphism
VAF: Variant allele fraction
scReQTL: Single cell RNA expressed quantitative trait loci
