# Supplementary material for: SCReadCounts: estimation of cell-level SNVs expression from scRNA-seq data
Source: BMC Genomics. 2021 Sep 22;22:689. doi: 10.1186/s12864-021-07974-8 (PMC8459565; doi:10.1186/s12864-021-07974-8)
Supplement: Supplementary file 1 — Additional file 1: Supplementary Fig. 1. CCND1. Two-dimensional UMAP clusters of samples SRR10156296, SRR10156297 and SRR10156299 showing cells classified by type (left) and visualizing COSV57121427 in CCND1, which was seen primarily in neurons in all three samples. The intensity of the red color corresponds to the proportion variant reads) of the VAFRNA of the indicated somatic mutations in the corresponding cells of the neuroblastoma samples; green color indicates that all the reads (minR = 3) covering the position in the cell carried the reference nucleotide. [file 12864_2021_7974_MOESM1_ESM.pdf]

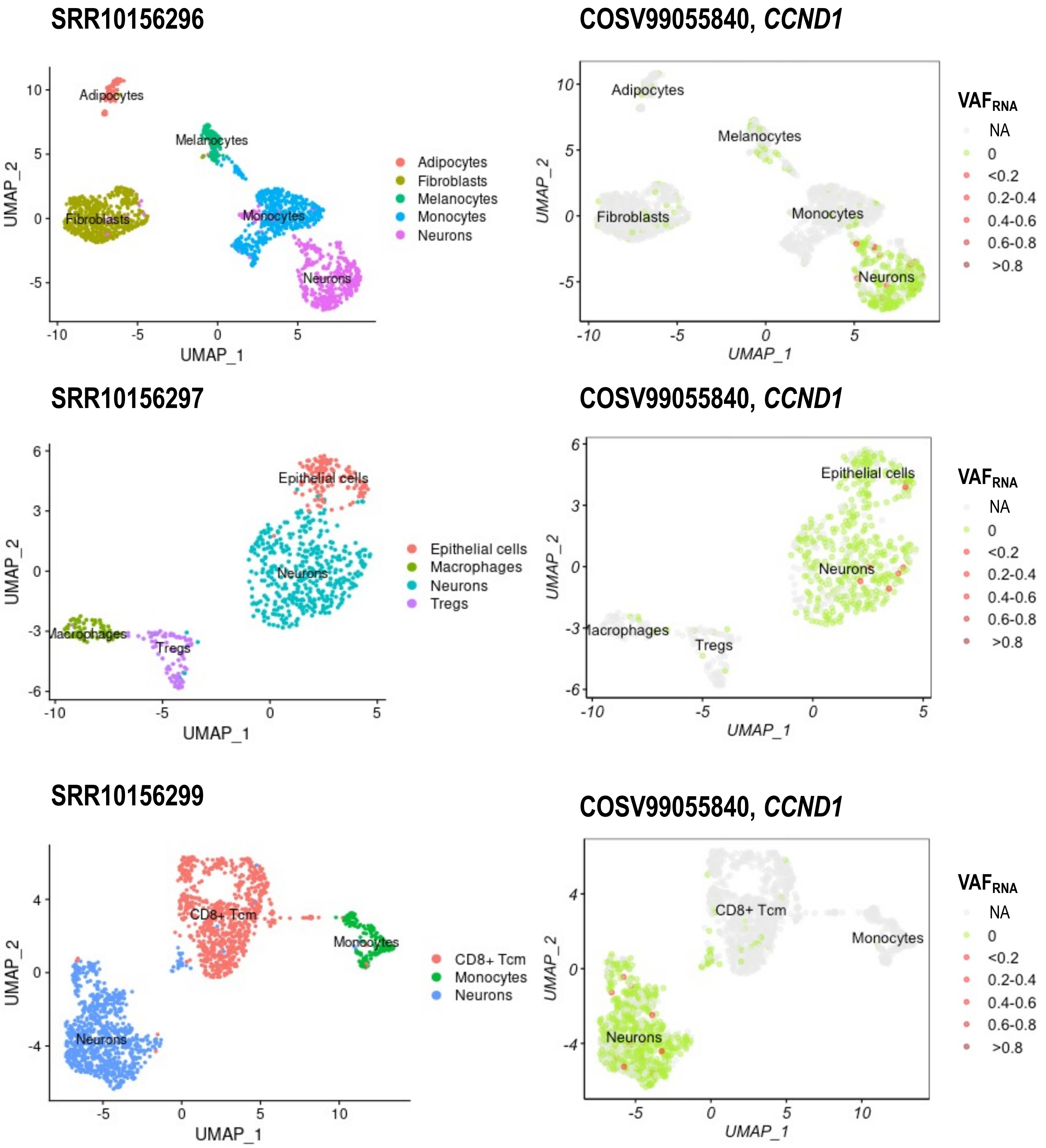

Supplementary Figure 1. Two-dimensional UMAP clusters of samples SRR10156296, SRR10156297 and SRR10156299 showing cells classified by type (left) and visualizing COSV57121427 in CCND1, which was seen primarily in neurons in all three samples. The intensity of the red color corresponds to the proportion variant reads) of the VAFRNA of the indicated somatic mutations in the corresponding cells of the neuroblastoma samples; green color indicates that all the reads (minR=3) covering the position in the cell carried the reference nucleotide.
