# Supplementary material for: SCReadCounts: estimation of cell-level SNVs expression from scRNA-seq data
Source: BMC Genomics. 2021 Sep 22;22:689. doi: 10.1186/s12864-021-07974-8 (PMC8459565; doi:10.1186/s12864-021-07974-8)
Supplement: Supplementary file 2 — Additional file 2: Supplementary Fig. 2. RNA-editing. Two-dimensional UMAP clusters of samples SRR10156297 and SRR10156299 showing cells classified by type (left) and visualizing RNA-editing levels (right) in the gene MEG3, where the intensity of the red color corresponds to the proportion of edited reads, and the green color indicates that all the reads (minR = 3) covering the position in the cell carried the reference nucleotide. [file 12864_2021_7974_MOESM2_ESM.pdf]

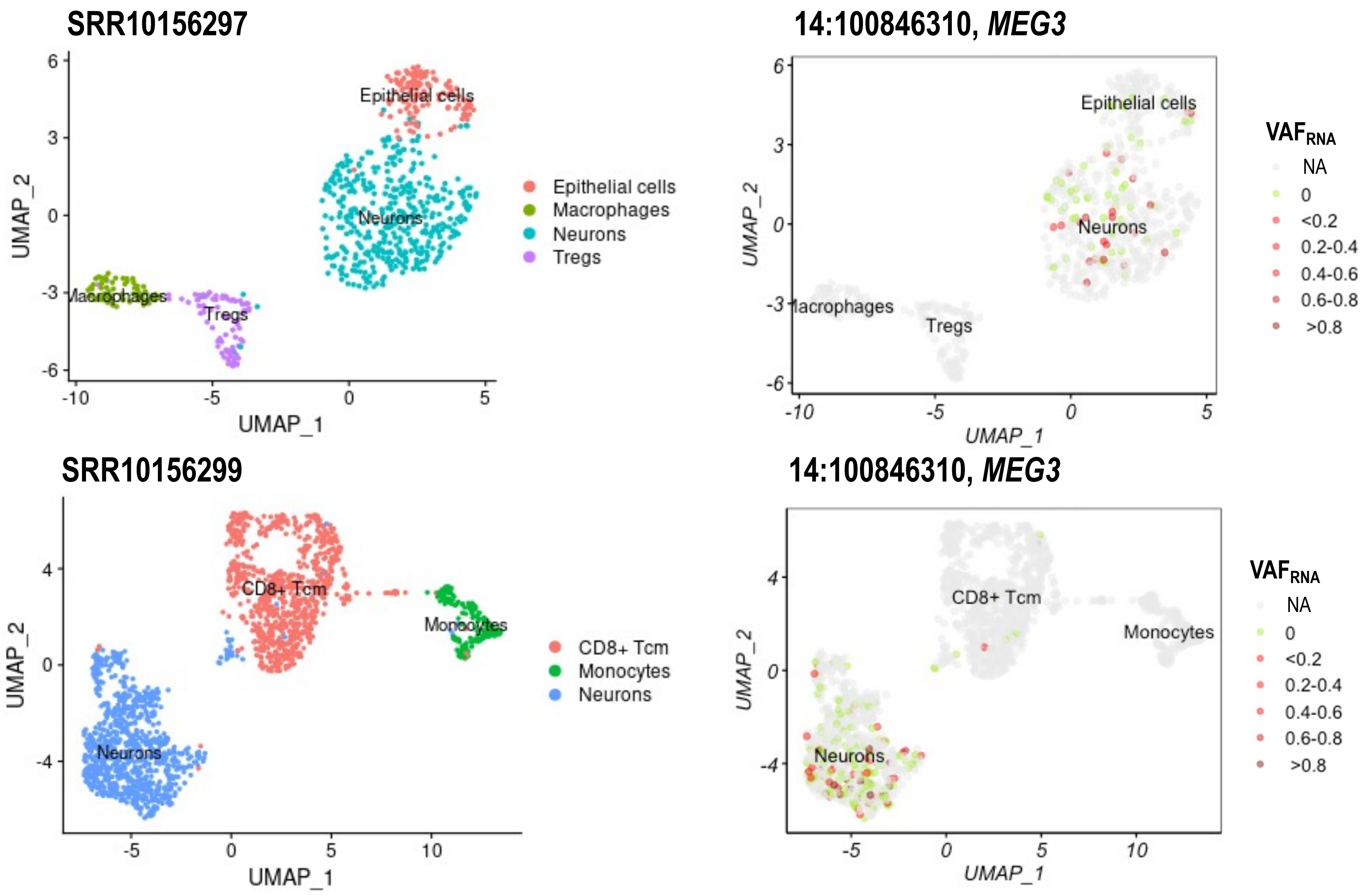

Supplementary Figure 2. Two-dimensional UMAP clusters of samples SRR10156297 and SRR10156299 showing cells classified by type (left) and visualizing RNA-editing levels (right) in the gene *MEG3*, where the intensity of the red color corresponds to the proportion of edited reads, and the green color indicates that all the reads (minR=3) covering the position in the cell carried the reference nucleotide.
