# Supplementary material for: SCReadCounts: estimation of cell-level SNVs expression from scRNA-seq data
Source: BMC Genomics. 2021 Sep 22;22:689. doi: 10.1186/s12864-021-07974-8 (PMC8459565; doi:10.1186/s12864-021-07974-8)
Supplement: Supplementary file 3 — Additional file 3: Supplementary Fig. 3. scVAFRNA distribution. scVAFRNA estimated at genomic positions covered by a minimum of 5 sequencing reads (minR = 5) at the sites with bi-allelic calls (GATK) in the 7 neuroblastoma samples; the positions are sorted by VAFRNA (y-axis). For the majority of positions, VAFRNA showed predominantly mono-allelic expression, with a substantial proportion of the scVAFRNA estimations in the intervals 0–0.2 (orange) and 0.8–1.0 (purple). The percentage of cells with the corresponding VAFRNA is displayed on the x-axis. [file 12864_2021_7974_MOESM3_ESM.pdf]

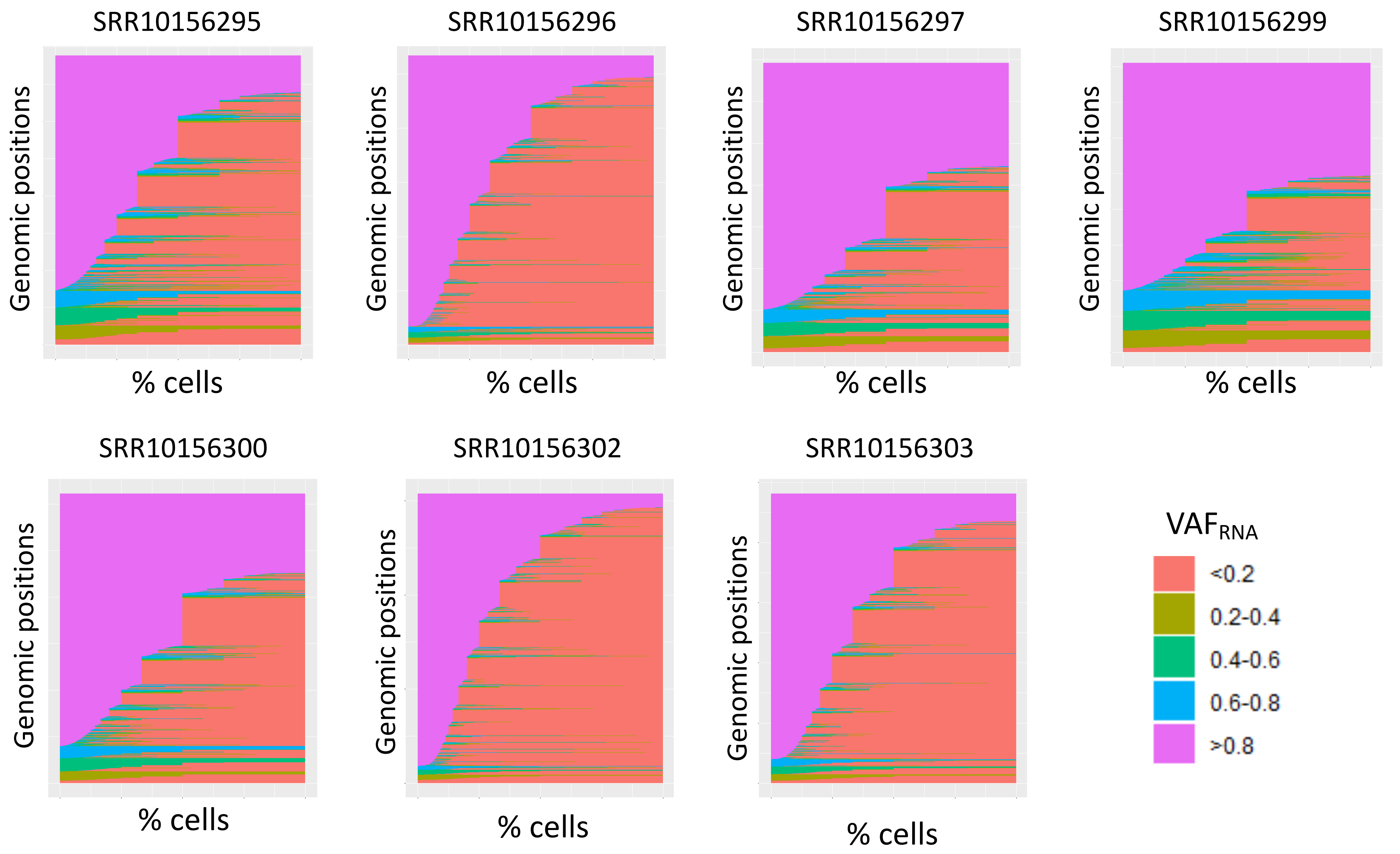

Supplementary Figure 3. scVAF$_{RNA}$ estimated at genomic positions covered by a minimum of 5 sequencing reads (minR=5) at the sites with bi-allelic calls (GATK) in the 7 neuroblastoma samples; the positions are sorted by VAFRNA (y-axis). For the majority of positions, VAF$_{RNA}$ showed predominantly mono-allelic expression, with a substantial proportion of the scVAF$_{RNA}$ estimations in the intervals 0-0.2 (orange) and 0.8-1.0 (purple). The percentage of cells with the corresponding VAFRNA is displayed on the x-axis.
