# Supplementary material for: SCReadCounts: estimation of cell-level SNVs expression from scRNA-seq data
Source: BMC Genomics. 2021 Sep 22;22:689. doi: 10.1186/s12864-021-07974-8 (PMC8459565; doi:10.1186/s12864-021-07974-8)
Supplement: Supplementary file 4 — Additional file 4: Supplementary Fig. 4. KRAS. IGV visualization of variable scVAFRNA of the novel somatic mutation Gly77Gly (12:25227293_G > A) in the gene KRAS in three individual cells of sample SRR10156295. [file 12864_2021_7974_MOESM4_ESM.pdf]

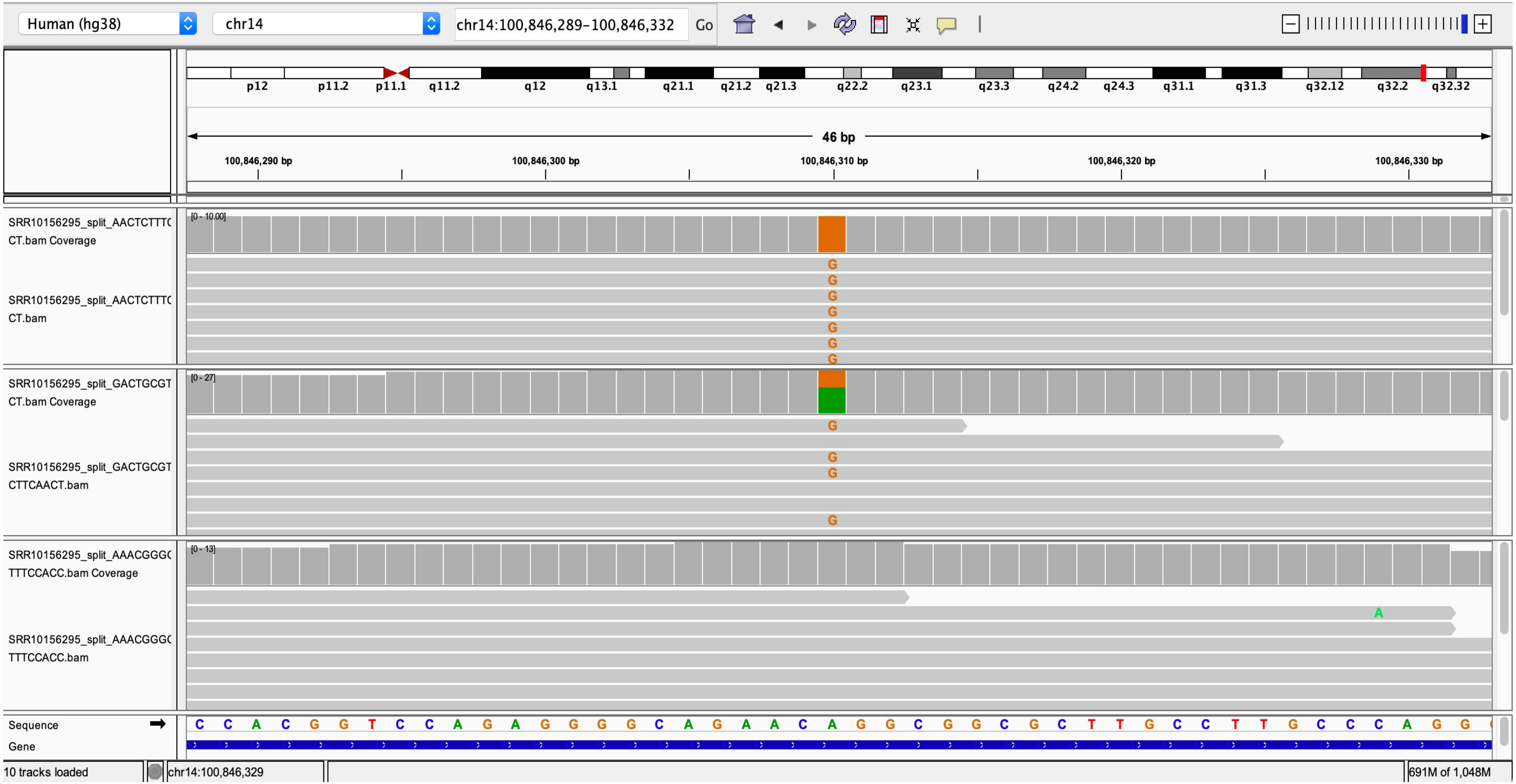

Supplementary Figure 4. IGV visualization of variable VAF_{RNA} of the novel somatic mutation Gly77Gly (12:25227293_G>A) in the gene *KRAS* in three individual cells of sample SRR10156295.
