# Supplementary material for: SCReadCounts: estimation of cell-level SNVs expression from scRNA-seq data
Source: BMC Genomics. 2021 Sep 22;22:689. doi: 10.1186/s12864-021-07974-8 (PMC8459565; doi:10.1186/s12864-021-07974-8)
Supplement: Supplementary file 5 — Additional file 5: Supplementary Fig. 5. Before and after filtering features distribution. Examples of density plots showing the distribution of cells based on proportion of transcripts of mitochondrial origin and number of genes, plotted against the counts of sequencing reads before (top) and after (bottom) filtering. The selected QC thresholds are: mitochondrial gene expression above between 6 and 15%, and number of genes below between 800 and 1000. To remove potential doublets/multiples we also filtered out signals with more than between 2600 and 4500 genes. [file 12864_2021_7974_MOESM5_ESM.pdf]

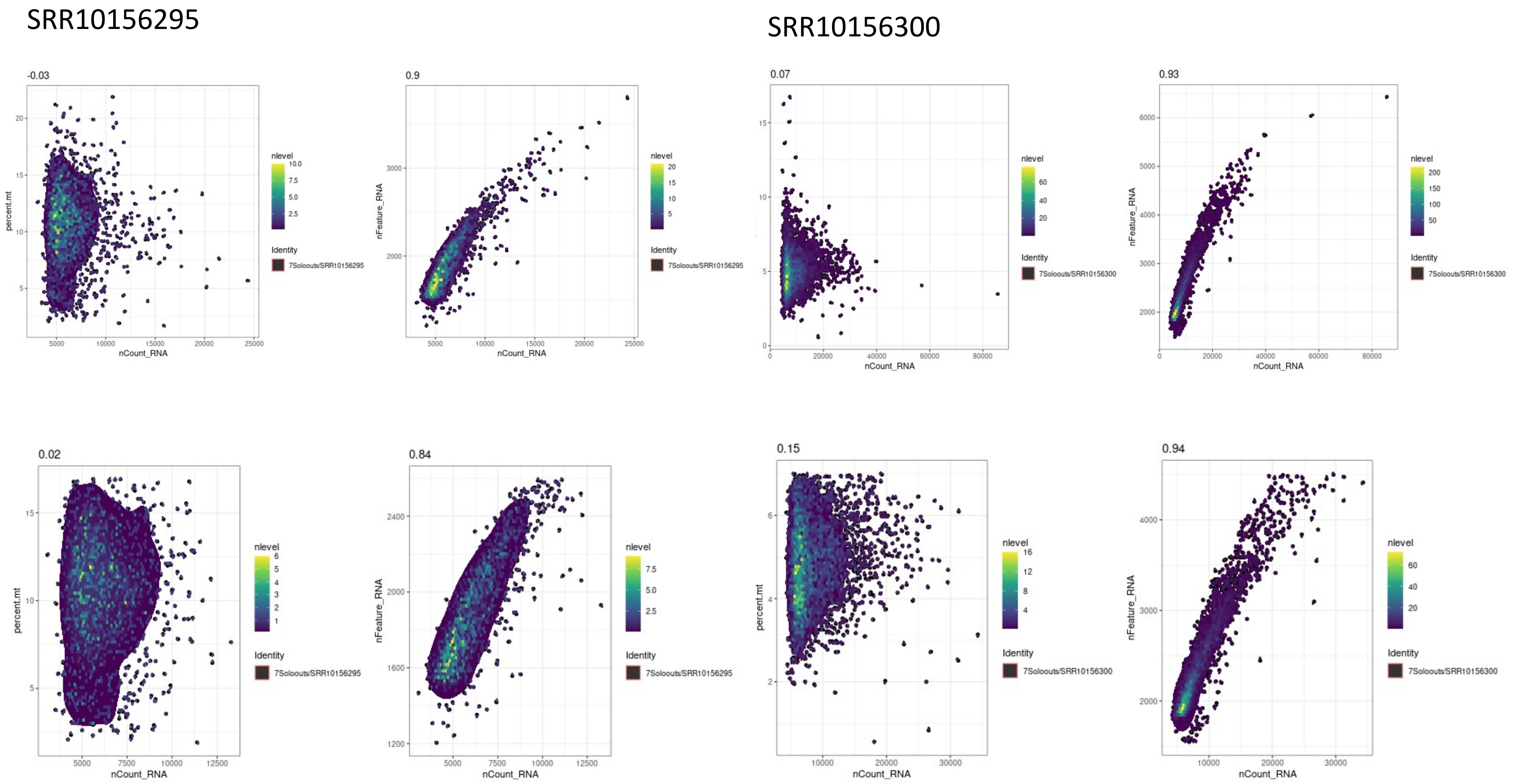

Supplementary Figure 5. Examples of density plots showing the distribution of cells based on proportion of transcripts of mitochondrial origin and number of genes, plotted against the counts of sequencing reads before (top) and after (bottom) filtering. The selected QC thresholds are: mitochondrial gene expression above between 6 and 15%, and number of genes below between 800 and 1000. To remove potential doublets/multiples we also filtered out signals with more than between 2600 and 4500 genes.
