# Supplementary material for: SCReadCounts: estimation of cell-level SNVs expression from scRNA-seq data
Source: BMC Genomics. 2021 Sep 22;22:689. doi: 10.1186/s12864-021-07974-8 (PMC8459565; doi:10.1186/s12864-021-07974-8)
Supplement: Supplementary file 6 — Additional file 6: Supplementary Fig. 6. SingleR Heatmaps. Heatmaps of SingleR scores for top correlated cell types from each of Seurat generated clusters. SingleR uses expression data to regenerate the clusters, and for each cluster, calculates the Spearman coefficient for the genes in the reference dataset. Then, it uses multiple correlation coefficient to collect a single value per cell type per cluster. [file 12864_2021_7974_MOESM6_ESM.pdf]

SRR10156295

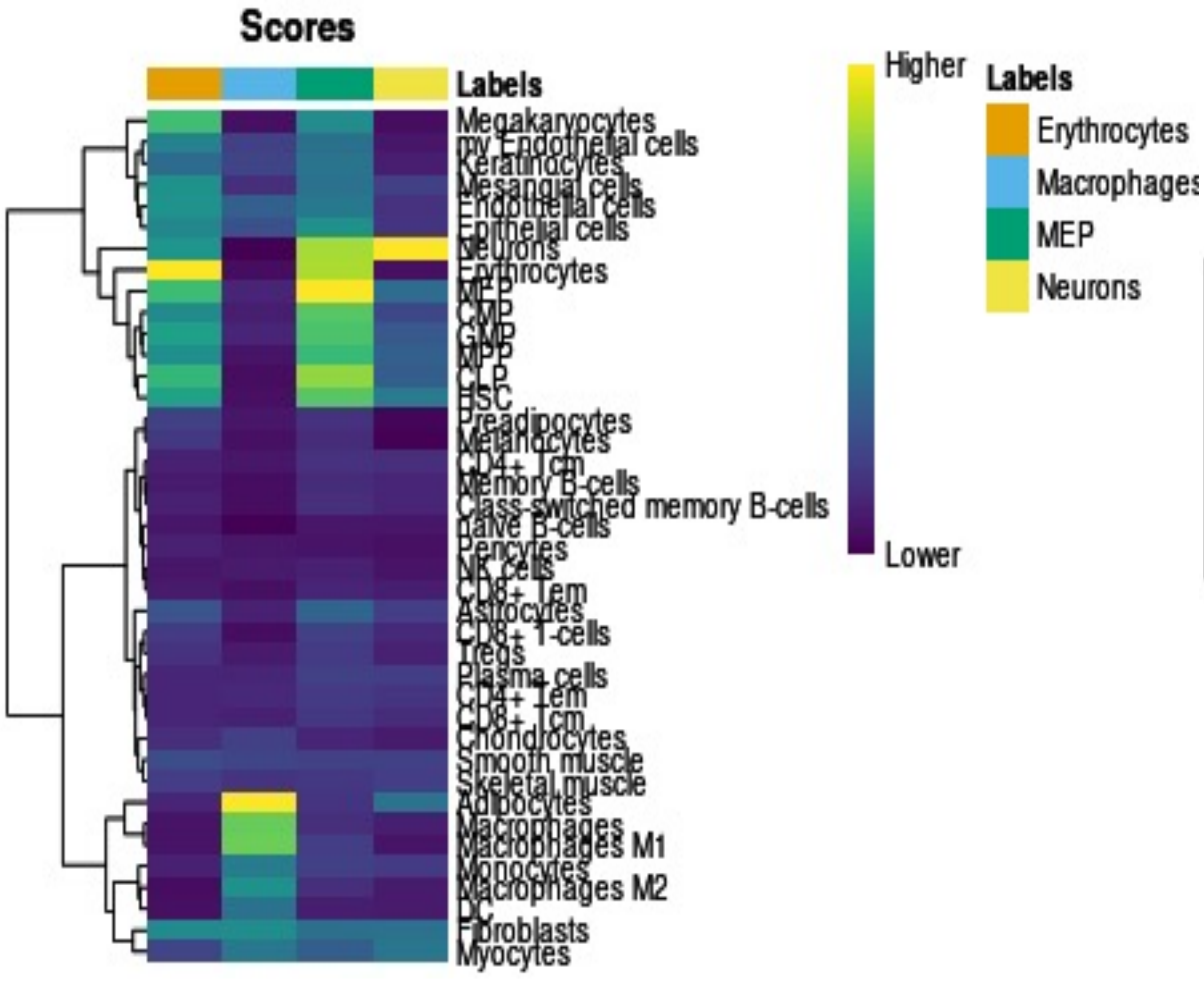

SRR10156297

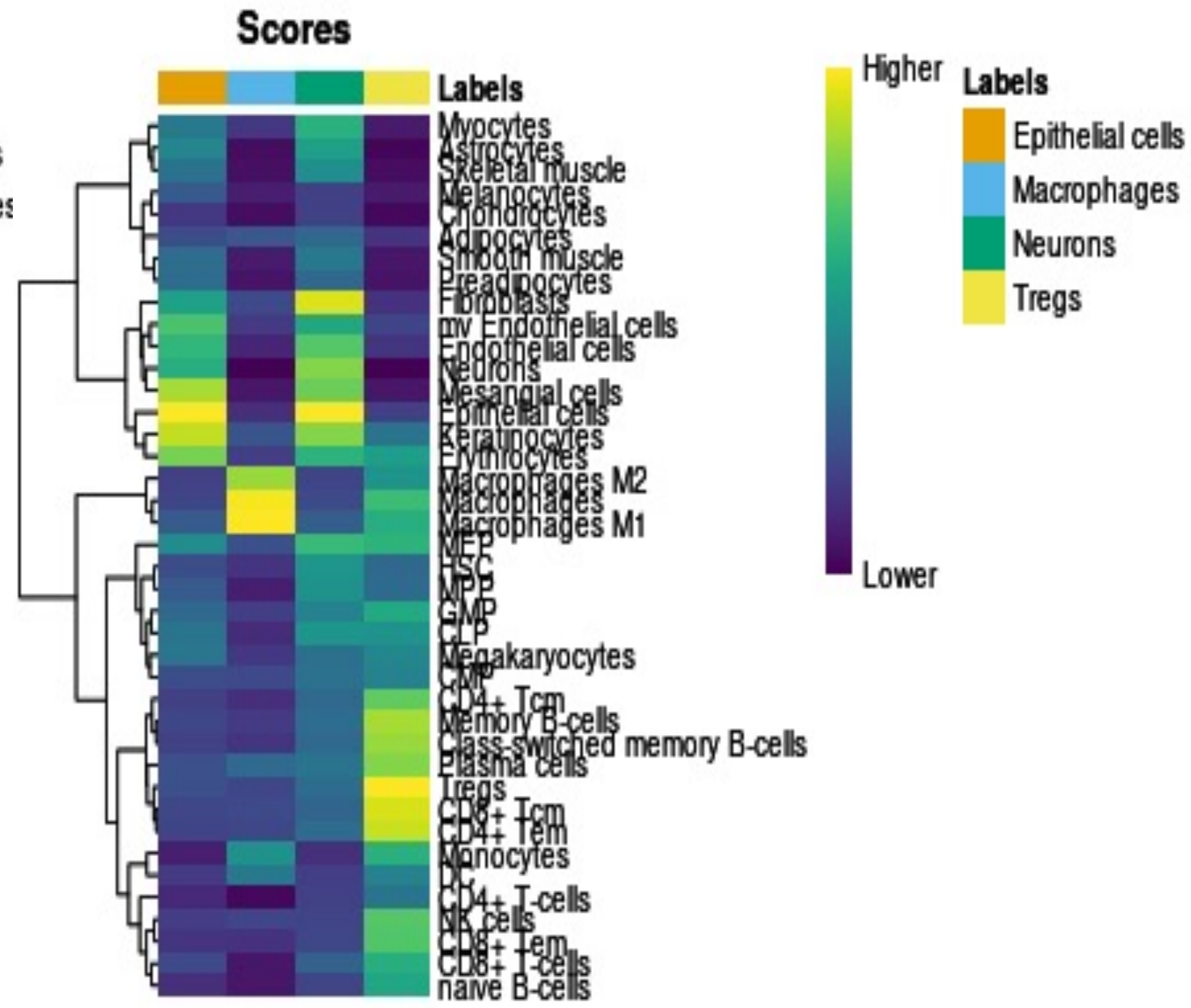

Supplementary Figure 6. Heatmaps of SingleR scores for top correlated cell types from each of Seurat generated clusters. SingleR uses expression data to regenerate the clusters, and for each cluster, calculates the Spearman coefficient for the genes in the reference dataset. Then, it uses multiple correlation coefficient to collect a single value per cell type per cluster.
